# Development of a Novel Positron Emission Tomography (PET) Radiotracer Targeting Bromodomain and Extra-Terminal Domain (BET) Family Proteins

**DOI:** 10.3389/fmolb.2020.00198

**Published:** 2020-08-12

**Authors:** Ping Bai, Yu Lan, Hao Wang, Zude Chen, Stephanie Fiedler, Robin Striar, Xiaoxia Lu, Changning Wang

**Affiliations:** ^1^Chengdu Institute of Biology, Chinese Academy of Sciences, Chengdu, China; ^2^Athinoula A. Martinos Center for Biomedical Imaging, Department of Radiology, Massachusetts General Hospital, Harvard Medical School, Boston, MA, United States; ^3^University of Chinese Academy of Sciences, Beijing, China

**Keywords:** epigenetic, bromodomain, PET, radiotracer, imaging

## Abstract

Bromodomain and extra-terminal domain (BET) family proteins have become a hot research area because of their close relationship with a variety of human diseases. The non-invasive imaging technique, such as positron emission tomography (PET), provides a powerful tool to visualize and quantify the BET family proteins that accelerating the investigation of this domain. Herein, we describe the development of a promising PET probe, **[**^11^**C]1**, specifically targeting BET family proteins based on the potent BET inhibitor CF53. **[**^11^**C]1** was successfully radio-synthesized with good yield and high purity after the optimization of radiolabeling conditions. The *in vivo* bio-activities evaluation of **[**^11^**C]1** was performed using PET imaging in rodents. The results demonstrated that **[**^11^**C]1** has favorable uptake in peripheral organs and moderate uptake in the brain. Further blocking studies indicated the high binding specificity and selectivity for BET proteins of this probe. Our findings suggest that **[**^11^**C]1** is a promising BET PET probe for BET proteins as well as epigenetic imaging.

## Introduction

The molecular imaging technique is one of the most advanced methods that has been used and integrated into various stages of the pipeline for biomedical research (Phelps, 2000; Ametamey et al., 2008). The utilization of the molecular imaging technique allows us to understand the biological mechanisms of proteins/receptors in a more comprehensive way than the traditional *in vitro* techniques. Among these, positron emission tomography (PET), a non-invasive imaging technique with high sensitivity as well as quantitation capability, is widely used in both clinical and research aspects. With a suitable imaging agent, PET can visualize the biomedical information and quantitatively measure the function of proteins and cellular processes in living subjects in a way that has never been done before (Alavi et al., 2004; Serdons et al., 2009). Therefore, the application of PET imaging into epigenetic research would be a great benefit for many aspects of this domain.

Bromodomain and extra-terminal domain (BET) family proteins are a class of epigenetic proteins that are distinguished from other bromodomain-containing proteins by the presence of two tandem bromodomains (BD1 and BD2) and one extra-terminal domain (ET) (Filippakopoulos et al., 2010; Filippakopoulos and Knapp, 2014; Smith and Zhou, 2016). Four subtypes (BRD2, BRD3, BRD4, and BRDT) of BET protein were identified in human and each of them has a distinctive role in epigenetic mechanisms. BRD4 binds to a variety of transcription factors and acts as a transcription facilitator in the transcription process to regulate cell growth. BRD3 and BRD2 can specifically interact with acetylated lysine in histones through their bromodomains and promote gene transcription. While BRDT, which is exclusively located in the testis, plays an essential role in spermatogenesis (Shang et al., 2004; LeRoy et al., 2008; Hay et al., 2014; Taniguchi, 2016). BET family proteins are thus known as epigenetic “readers” that regulate the gene transcription and epigenetic process (Conway et al., 2015).

With the intensive studies on the physiological mechanism of BET proteins, it was found that they are involved in the occurrence and development of a range of human diseases including cancer, inflammation, and neurodegeneration et al. (Doroshow et al., 2017; Pervaiz et al., 2018; Cochran et al., 2019; Faivre et al., 2020). Subsequently, BET family proteins become a promising therapeutic target that attracting researchers’ attention. Over the decades, the development of novel small-molecule BET inhibitor has become a focus of attention in both academic and pharmaceutical research since the first potent BET inhibitor, JQ1, was reported (Filippakopoulos et al., 2010; Brand et al., 2015; Doroshow et al., 2017). For example, a series of BET inhibitors including I-BET726, OTX015, ABBV-075, and INCB054329 were discovered in succession and are currently under further clinical development (Garnier et al., 2014; Gosmini et al., 2014; Berthon et al., 2016; Bui et al., 2017; Wilson et al., 2018). These small-molecule compounds were found to competitively inhibit BET proteins by binding with the acetylated lysine residues in the histones that regulating the epigenetic mechanism and leading to a therapeutic effect on a variety of diseases. The emergence of BET inhibitors provides a powerful tool for the investigation of BET family proteins in the epigenetic mechanism and even an alternative treatment strategy for many diseases. However, many basic questions remain unanswered and the role of BET proteins in physiology and pathology are still in need of further study.

**FIGURE 1:**
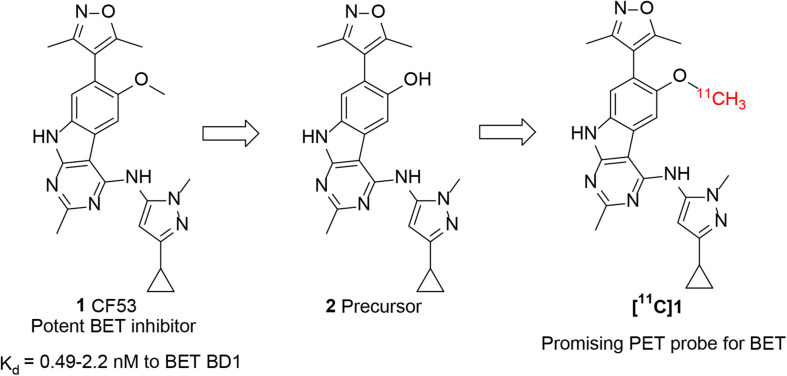
Chemical structure of **1** (CF53), **2** (precursor), and **[**^11^**C]1**.

For accurate visualization and quantification of BET proteins by PET imaging, it is critical to develop a suitable PET radiotracer that specifically targets BET proteins. However, there are no PET tracers that can image BET family proteins in human. To address this challenge, our investigation started with screening the chemotypes of BET inhibitors in the available literatures, with a goal of radiolabeling with isotope such as C-11 or F-18 that apply them to the BET proteins imaging. Compound **1** (CF53, Figure 1) is a potent BET inhibitor that has a high binding affinity with K_*i*_ value of <1 nM to BET BD1 as well as high selectivity over non-BET bromodomain-containing proteins (Zhao et al., 2018). After structural analysis, we first obtained the precursor (**2**) through the demethylation of **1** and radiolabeled **1** as a BET PET radioligand (Figure 1). The PET/CT imaging in rodents was then performed to evaluate the potential of **[**^11^**C]1** to be a non-invasive tool for BET protein quantification and epigenetic research.

## Results and Discussion

### Synthesis of BET PET Imaging Agent

**SCHEME 1:**
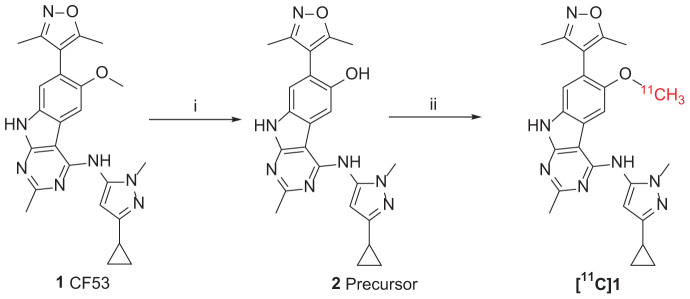
Synthesis of Precursor **2** and **[**^11^**C]1**. Reagents and conditions: (i) BBr_3_ (1.0 mol/L in dichloromethane), dichloromethane, −78°C to room temperature, overnight; (ii) **2** (precursor, 0.5 mg), [^11^C]CH_3_I, NaOH (1 M. 10 μL), in 0.3 mL DMF, 3 min, 80°C. Radiochemical yield (RCY): 20–25% (non-decay corrected to trapped [^11^C]CH_3_I).

The synthesis of the precursor was achieved via demethylation of **1** using boron tribromide with a high yield (Scheme 1). Subsequently, trapped [^11^C]CH_3_I was used for reacting with the exposed hydroxyl group of **1** in the presence of a suitable base to obtain **[**^11^**C]1**. Nevertheless, three methylation sites were found on precursor **2**, two secondary amine groups and one hydroxyl group, that may afford a mixture of the N-methylated and O-methylated radioactive products when treating with [^11^C]CH_3_I and base. To ensure a good radiochemical yield (RCY) of **[**^11^**C]1** for animal study, the methylation conditions for the “cold” reaction were screened using stoichiometric amounts of CH_3_I (1 eq. in solution), precursor **2** (1 mg), and several bases at different temperatures.

The reaction was first tested under mildly alkaline condition using K_2_CO_3_ as the base at 100°C. It was found that the N-methylation products were predominantly formed and the yield of the O-methylation product was very low. When the reaction temperature decreased from 100 to 80°C and using the stronger base NaOH/KOH, the ration of O-methylation was increased. To improve the yield of O-methylation and enable separating the desired products by the reverse-phase semipreparative HPLC, we further optimized the reaction condition by screening bases and temperatures. As is shown in Table 1, comparing with the temperature, alkali has a greater effect on the N/O-methylation and the largest proportion of O-methylated products was observed in the condition of 1M NaOH at 80°C, which we thus used it for the **[**^11^**C]1** preparation.

**TABLE 1:** Assessment of N-methylation to O-methylation selectivity for various reaction conditions.

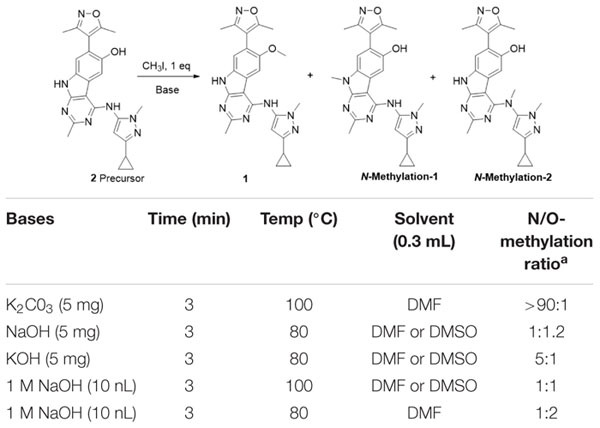				

*a The yield was measured by HPLC.*

**[**^11^**C]1** was achieved according to the synthetic route presenting in Scheme 1. The [^11^C]CH_3_I was produced form the cyclotron and bubbled through a solution of precursor **2** and 1 M NaOH in DMF at room temperature. The [^11^C]CH_3_I was trapped and the mixture was stirred for 3 min at 80°C. The radioactive product was then purified by semi-preparative reversed-phase HPLC and reformulated to obtain **[**^11^**C]1** with good RCY and high purity (RCY = 10-15%, non-decay corrected to trapped [^11^C]CH_3_I; purity >95%, *n* = 3) (Supplementary Figure S1 in supporting information). Including formulation, the total preparation of **[**^11^**C]1** was taken for 35–40 min after the end of bombardment (EOB) with average molar radioactivity of 352 GBq/μmol.

### Rodents PET/CT Imaging

**FIGURE 2:**
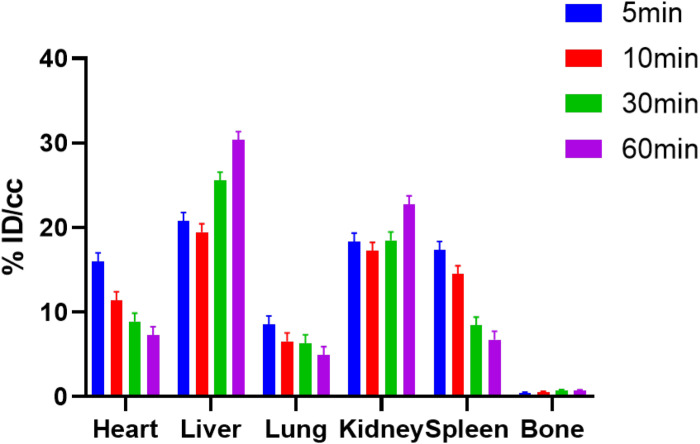
The bio-distribution of **[**^11^**C]1** in mice at 5, 10, 30, and 60 min postinjection in mice (*n* = 2 for each time point).

Following the successful radiosynthesis, we stepped forward to evaluate **[**^11^**C]1** as a potential BET PET radiotracer by PET imaging experiments in rodents. Dynamic PET imaging in male Balb/c mice was conducted following the injection of **[**^11^**C]1** via the tail vein at the dose of 3.7–7.4 Mbq for each scan. Each dynamic PET imaging scan lasted for 60 min and followed by a 10-min of computed tomography (CT) scan. The PET/CT imaging analysis was then performed by dynamic data collection and image reconstruction. The biodistribution of **[**^11^**C]1** in the whole-body of mice is initially studied. As depicted in Figure 2, four time-points (5, 10, 30, and 60 min) after **[**^11^**C]1** injection were selected to evaluate the uptake, biodistribution, and clearance of the radioligand in the organs of interests. The histogram revealed that **[**^11^**C]1** was highly distributed in the blood-rich organs such as the heart, liver, and kidney. In the heart, spleen, and lung, the uptakes of radioligand reached a peak sharply and followed by gradual wash-out post-injection. While in the liver and kidney, the radioactivity level rose rapidly in the first few minutes and accumulated gradually after a slight drop at approximately 10 min post-injection, which indicated that **[**^11^**C]1** was possibly eliminated via the hepatobiliary and urinary pathway.

**FIGURE 3:**
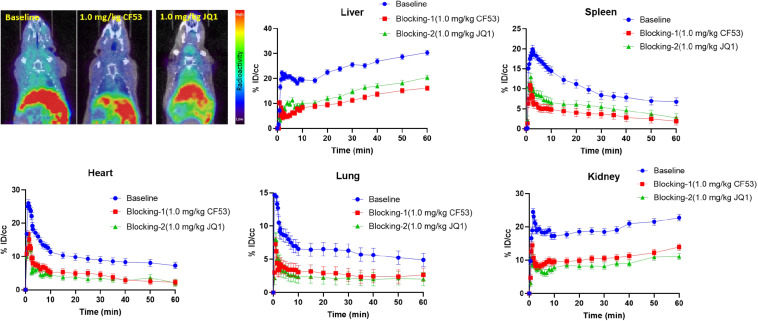
The mice body PET/CT baseline and blocking (1.0 mg/kg CF53 and 1.0 mg/kg JQ1) imaging of **[**^11^**C]1** and time-activity curve of organs of interest demonstrating uptake of radioligand for baseline and blocking studies (The images are summed from 20 min to 60 min, *n* = 2).

To evaluate the specific binding of **[**^11^**C]1**, the blocking studies were carried out. Mice were pre-treated with unlabeled **1** (1.0 mg/kg) and JQ1 (1.0 mg/kg) at 5-min prior to the administration of **[**^11^**C]1**. The PET/CT imaging and time-activity curves (TAC) of baseline and blocking studies are shown separately in Figure 3. Of note, the uptake of **[**^11^**C]1** in the regions of interest was significantly decreased in both self-blocking and JQ1-blocking experiments. The concentration of radioligand was decreased by over 40% on average in organs of interest suggesting the excellent specific binding of **[**^11^**C]1** [the radioactivity was expressed as the percentage of injected dose per cubic centimeter (% ID/cc) and the uptake changes were calculated by the radioactivity percent change between maximum uptake and the minimum uptake in selected organs].

**FIGURE 4:**
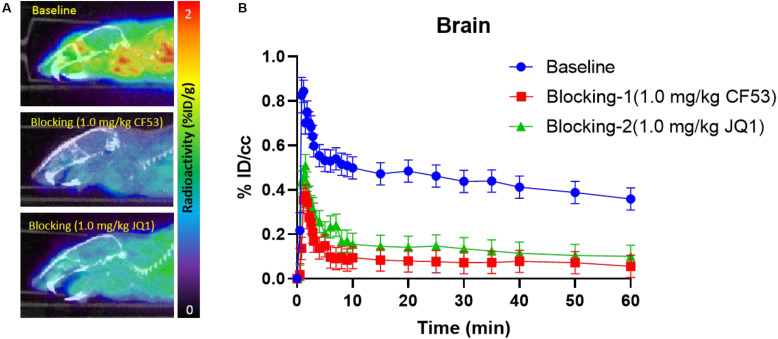
**(A)** The mice brain PET/CT imaging of **[**^11^**C]1** focuses on the brain and the baseline and blocking (1.0 mg/kg CF53 and 1.0 mg/kg JQ1) **(B)** time-activity curves of **[**^11^**C]1** (The images are summed from 20 min to 60 min, *n* = 2).

Encouraged by the promising results in the peripheral regions, we further investigated the bio-characteristics of **[**^11^**C]1** in the brain (Figure 4). Based on a whole-brain analysis, moderate brain uptake with a max % ID/cc value of 0.9 at ∼2 min post-injection was found, indicating the limited brain-blood barrier (BBB) penetration of this radiotracer. The BBB penetration ability for a radioligand could be determined by some important physicochemical properties such as molecular weight (M.Wt), lipophilicity (logP/logD), and total polar surface area (tPSA). According to our previous experience, the physicochemical properties of a PET imaging probe with high BBB permeability are preferred for M. Wt < 500, CLogP ≤ 4, and tPSA between 30 and 75 (Bai et al., 2020b). The M. Wt (443.5) and CLogP (3.1) of **[**^11^**C]1** are in the range of preferred values, while the relative large tPSA (95.2) of **[**^11^**C]1** may lead to the limitation of BBB penetration (the M.Wt, CLogP, and tPSA of **1** were calculated by ChemDraw 14.0). Despite the moderate brain uptake level of **[**^11^**C]1** makes it less sensitive to support the BET family proteins imaging in the central nervous system (CNS), the distinct reduction of radioactivity uptake was observed in the blocking studies. Based on the TACs, approximate 50% radioactivity reduction in the whole-brain in the self-blocking and JQ1-blocking studies, demonstrating the high specific binding of **[**^11^**C]1**.

## Conclusion

In summary, a PET radiotracer, **[**^11^**C]1**, specifically targets BET family proteins was developed based on the reported BET inhibitor CF53. We screened the radiolabeling conditions in “cold” reactions to optimize the RCY. After successfully radiosynthesis of the radioligand with good yield and high purity, the *in vivo* evaluation was performed using PET imaging in rodents. The biodistribution demonstrated that **[**^11^**C]1** has favorable uptake in peripheral organs and moderate uptake in the brain. Further blocking studies with unlabeled **1** and JQ1 pretreatment in the rodents indicated the high specific binding of **[**^11^**C]1**. In conclusion, **[**^11^**C]1** is a promising BET PET probe for epigenetic imaging and further investigation is expected.

## Materials and Methods

The purity of all final compounds was determined by HPLC analysis and showed purity >95%. Compound **1** (CF53) was purchased form MedChemExpress; anhydrous dimethylformamide (DMF) was purchased from Acros Organics. The analytical separation was conducted on an Agilent 1100 series HPLC fitted with a diode-array detector, quaternary pump, vacuum degasser, and autosampler. Mass spectrometry data were recorded on an Agilent 6310 ion trap mass spectrometer (ESI source) connected to an Agilent 1200 series HPLC with a quaternary pump, vacuum degasser, diode-array detector, and autosampler.

[^11^C]CO_2_ was produced via the ^14^N (p, α) ^11^C reaction on nitrogen with 2.5% oxygen, with 11 MeV protons (Siemens Eclipse cyclotron), and trapped on molecular sieves in a TRACERlab FX-MeI synthesizer (General Electric). [^11^C]CH_4_ was obtained by the reduction of [^11^C]CO_2_ in the presence of Ni/hydrogen at 350°C and recirculated through an oven containing I_2_ to produce ^11^CH_3_I via a radical reaction.

All animal studies were carried out at Massachusetts General Hospital (PHS Assurance of Compliance No. A3596-01). The Subcommittee on Research Animal Care (SRAC) serves as the Institutional Animal Care and Use Committee (IACUC) for the Massachusetts General Hospital (MGH). SRAC reviewed and approved all procedures detailed in this paper.

### Synthesis of 4-((3-cyclopropyl-1-methyl-1H-pyrazol-5-yl)amino)-7-(3,5-dimethylisoxazol-4-yl)-2-methyl-9H-pyrimido[4,5-b]indol-6-ol (2, precursor)

To a solution of **1** (CF53, 20 mg, 0.045 mmol) in dichloromethane (5 mL) was carefully added the boron tribromide solution (0.45 mL, 1.0 mol/L in dichloromethane) at −78°C under N_2_. The resulting mixture was gradually warmed to room temperature and left standing overnight. After complete consumption of compound **1**, an additional 20 mL of H_2_O and 20 mL of dichloromethane were carefully added to the reaction mixture successively. The organic layer was separated and the aqueous layer was extracted with dichloromethane (100 mL × 4). the organic phases were combined and washed with brine, dried over MgSO_4_, filtered, and concentrated *in vacuo*. The crude product was purified using flash chromatography (dichloromethane/methanol = 10/1) to yield the 4-((3-cyclopropyl-1-methyl-1H-pyrazol-5-yl)amino)-7-(3,5-dimethylisoxazol-4-yl)-2-methyl-9H-pyrimido[4,5-b]indol-6-ol (**2**, precursor) as a light yellow solid (11 mg, 57.9%). LC–MS Calcd. for C_23_H_23_N_7_O_2_ expected [M + H]^+^: 430.2; Found [M + H]^+^: 430.2.

### Radiosynthesis of [^11^C]1

The precursor (**2**) was dissolved in a solution of anhydrous DMF (1.0 mg/mL, 0.3 mL) and NaOH (1 M, 10 μL). [^11^C]CH_3_I was produced from the cyclotron and bubbled through the precursor-containing mixture for 3 min at room temperature. The mixture was stirred at 80°C for 3 min, and then cool down at room temperature. The reaction mixture was quenched by a 1.2 mL HPLC mobile phase and then injected into a reverse-phase semipreparative HPLC for purification. The HPLC purification was carried out on a column of Agilent Eclipse XDB-C18 (5 μm, 250 mm × 9.4 mm), with a mobile phase of 0.1% TFA in water/0.1% TFA in acetonitrile (70/30, *v*/*v*) at a flow rate of 5.0 mL/min. The desired product was collected and reformulated by loading onto a solid-phase exchange (SPE) C-18 Sep-Pak cartridge, rinsing with H_2_O (5 mL), eluting with ethanol (1 mL), and diluting with saline solution (0.9%, 9 mL). The average synthesis time was 35–40 min from the end of cyclotron bombardment. The average RCY was 10-15% (non-decay corrected to trapped [^11^C]CH_3_I) and purity >95%. The molar radioactivity of **[**^11^**C]1** was determined by the HPLC comparison of UV absorbance at 254 nm with concentrations of **1**.

### Rodent PET/CT Acquisition and Post Processing

The general procedure for rodent PET/CT studies was described previously (Bai et al., 2019, 2020a). Briefly, the PET/CT scan was carried out by a Triumph Trimodality/SPECT scanner (Gamma Medica, Northridge, CA, United States). Mice were arranged under anesthetized 2% isoflurane (Patterson Vet Supply, Inc., Greeley, CO, United States) in a carrier of 2 L/min medical oxygen during the imaging scanning. **[**^11^**C]1** (3.7–7.4 Mbq per animal, *n* = 2) was injected in mice via a lateral tail vein catheterization before PET acquisition. For the blocking study, the radioligand was administrated after CF53, or JQ1 was injected via a lateral tail vein catheterization 5-min before the start of PET acquisition. Dynamic PET acquisition lasted for 60 min followed by 10-min CT for anatomic co-registration. PET data were reconstructed using a 3D-MLEM method resulting in full width at a half-maximum resolution of 1 mm. These files were imported and analyzed using AMIDE (Loening and Gambhir, 2003) (a medical imaging data examiner) software (an open-source software, Los Angeles, CA, United States) and PMOD (PMOD 4.01, PMOD Technologies Ltd., Zurich, Switzerland).

### Rodent PET/CT Image Analysis

Dynamic PET data were collected and the corresponding images were reconstructed by 3D-MLEM method resulting in full width at a half-maximum resolution of 1 mm. Volumes of interest (VOIs) were generated manually in forms of spheres under the guide of high-resolution CT structural images. TACs were exported as decay-corrected activity per unit volume. The TACs were expressed as percent injected dose per unit volume (%ID/cc) for analysis.

## Data Availability Statement

The datasets presented in this study can be found in online repositories. The names of the repository/repositories and accession number(s) can be found in the article/Supplementary Material.

## Ethics Statement

The animal study was reviewed and approved by the IACUC committee at Massachusetts General Hospital.

## Author Contributions

PB, YL, HW, and ZC performed the research. PB, SF, RS, and XL contributed to writing and revising the manuscript. All authors read and approved the manuscript.

## Conflict of Interest

The authors declare that the research was conducted in the absence of any commercial or financial relationships that could be construed as a potential conflict of interest.
