# Supplementary material for: Development of a Novel Positron Emission Tomography (PET) Radiotracer Targeting Bromodomain and Extra-Terminal Domain (BET) Family Proteins
Source: Front Mol Biosci. 2020 Aug 12;7:198. doi: 10.3389/fmolb.2020.00198 (PMC7434981; doi:10.3389/fmolb.2020.00198)
Supplement: Supplementary file 1 [file Image_1.pdf]

Supplementary Material

Development of a Novel Positron Emission Tomography (PET) Radiotracer Targeting Bromodomain and Extra-Terminal Domain (BET) Family Proteins

Ping Bai[1,2,3], Yu Lan[2], Hao Wang[2], Zude Chen[2],Stephanie Fiedler[2], Robin Striar[2], Xiaoxia Lu[1*],Changning Wang[2*]

[1]Chengdu Institute of Biology, Chinese Academy of Sciences, Chengdu 610041, PR China

[2]Athinoula A. Martinos Center for Biomedical Imaging, Department of Radiology, Massachusetts General Hospital, Harvard Medical School, Charlestown, MA 02129, USA.

[3]University of Chinese Academy of Sciences, Beijing 100049, PR China

*To whom correspondence should be addressed:

Changning Wang, PhD

Martinos Center for Biomedical Imaging at Massachusetts General Hospital, Harvard Medical School

149 13th Street, Suite 2301

Charlestown, MA 02129

617-724-3983

cwang15@mgh.harvard.edu

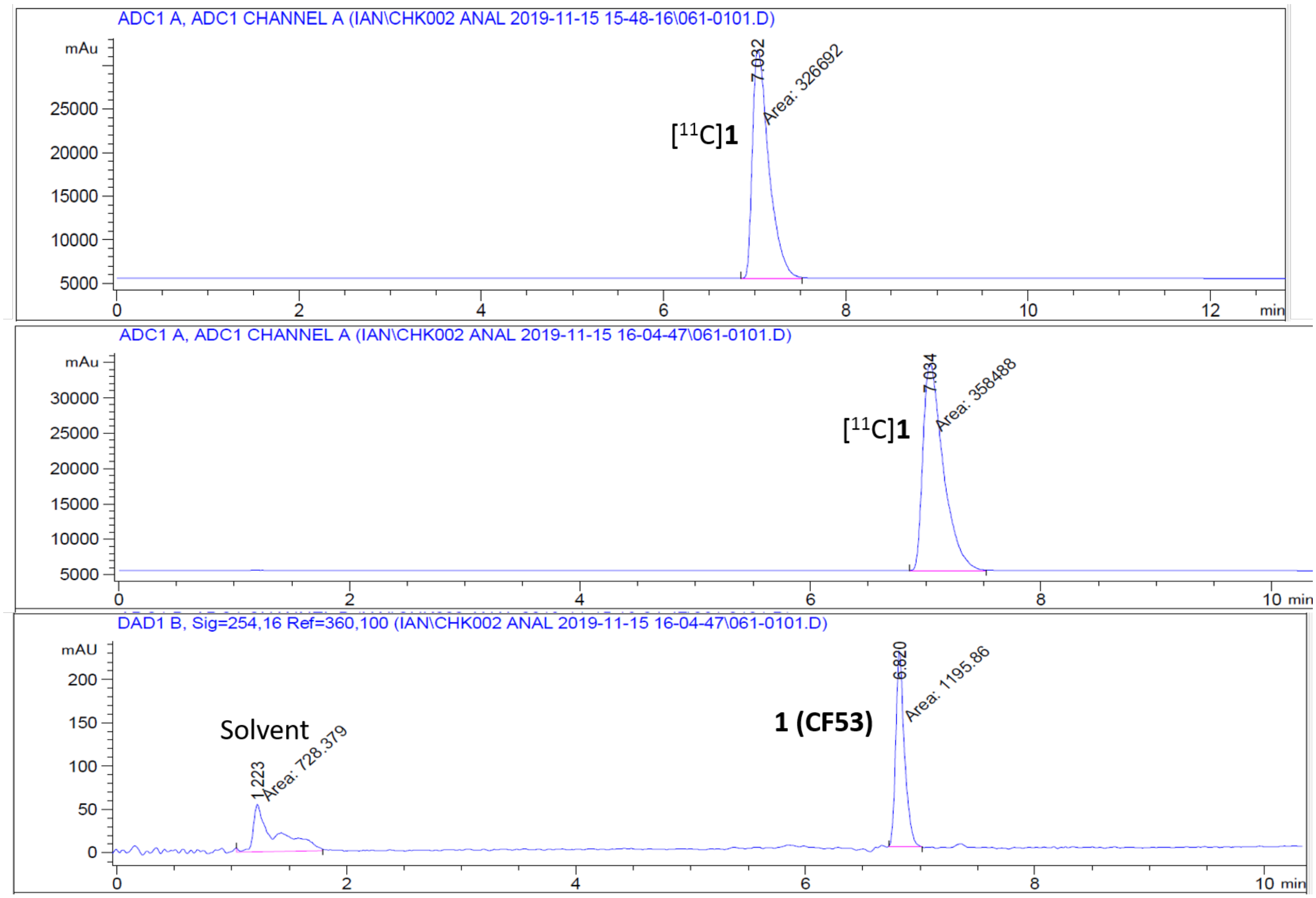

Supplementary Figure S1. The HPLC chromatogram of **[^{11}C]1** and the co-injection with standard **1** (CF53).
